# Chronic Penile Pain: A Poorly Researched and Managed Life-Debilitating Condition

**DOI:** 10.7759/cureus.49776

**Published:** 2023-12-01

**Authors:** Jing Huang, Nishma Shah, Rachel Bailon, Sabrina Trammel

**Affiliations:** 1 College of Medicine, Nova Southeastern University Dr. Kiran C. Patel College of Osteopathic Medicine, Fort Lauderdale, USA; 2 Family Medicine, Stony Brook University, Southampton, USA

**Keywords:** sacral neuromodulation, interstim device, chronic penile pain, pudendal neuropathy, overactive bladder

## Abstract

Chronic penile pain is a complex clinical entity with limited diagnostic criteria and treatment options. Due to limited reporting of these cases, there are no clear clinical treatments and indications for when these patients present to the clinic. This case report will highlight the diagnostic challenges encountered and the subsequent management strategies employed while working up a patient with penile pain.

We present a 37-year-old male with a six-year history of debilitating penile pain, urinary frequency, and urgency that is exacerbated by sexual arousal and touch. Initial evaluations attributed the symptoms to medication side effects, leading to medication changes. Despite multiple treatments, including gabapentin, solifenacin, vibegron, and a variety of specialist consultations, the patient's condition persisted. Neurological evaluation revealed pudendal neuropathy. Medical management with pudendal nerve blocks and gabapentin did not provide lasting relief, so surgical interventions were considered. Subsequent treatment with an InterStim II device (Medtronic Inc., Minneapolis, MN) initially resulted in significant symptom improvement. Unfortunately, at the seven-month follow-up, his pain returned. Further evaluation and additional treatment options are currently under consideration. This case report highlights the diagnostic complexity and limited treatment options for chronic penile pain. It suggests that sacral neuromodulation, although lacking long-term data, may offer temporary relief in cases refractory to medical therapy. Further research is needed to enhance our understanding and management of this challenging condition.

## Introduction

Patients presenting to the office with chronic urogenital pain can be difficult for clinicians to diagnose and manage due to the variability of presenting symptoms [1]. Pinpointing a specific visceral cause of penile pain is challenging due to the potential of referral pain [1]. Chronic pelvic pain (CPP) can be classified as pelvic or non-pelvic in origin and can stem from many systems including but not limited to muscular, neurologic, urologic, and/or anorectal [1,2]. Penile pain, a subcategory of pelvic pain syndrome, is infrequently reported in the literature, despite the considerable impact that penile pain imposes on patients' sexual and mental health. Treatment of penile pain is typically geared toward the treatment of the underlying cause (priapism, Peyronie’s disease, and dorsal nerve compression) [3,4]. While various pharmacological and surgical management modalities have been explored, their long-term efficacies remain uncertain [5,6]. Subcutaneous steroid injections are effective in treating patients with penile pain associated with Peyronie’s disease [5]. A case reported by Wordekemper et al. suggested that CPP refractory to medication may be treated with cryoablation [6]. However, both managements have provided only temporary relief, with steroid injections having a mean pain-free period of 23.8 months and cryoablation for four months [5,6]. This case report aims to present a comprehensive clinical account of a patient with chronic penile pain, highlighting the diagnostic challenges encountered and the management strategies employed.

## Case presentation

A 37-year-old male with a past medical history of chronic penile pain, overactive bladder, mild obstructive sleep apnea, hypothyroidism, anxiety, depression, insomnia, varicose veins, and learning disability presented with a six-year history of penile pain and urinary frequency. The pain was described as a "pressure-like" sensation associated with urinary frequency and urgency. The pain was primarily located at the glans and exacerbated by sexual arousal and touch. In some instances, the pain was debilitating.

Before the onset of his penile symptoms, he was diagnosed with anxiety and depression and placed on sertraline. Urology was consulted, who determined that the symptoms were due to medication side effects. He was then placed on ibuprofen, which temporarily alleviated the penile pressure. It was suspected that sertraline was causing this new sensation, so the patient was transitioned to quetiapine and later alfuzosin without symptom improvement. He continued to have penile pressure, unrelieved with masturbation or urination, that awoke him from sleep, associated with urinary frequency and urgency. His pain was then suspected to be neuropathic in nature, and he was started on gabapentin.

**Figure 1:**
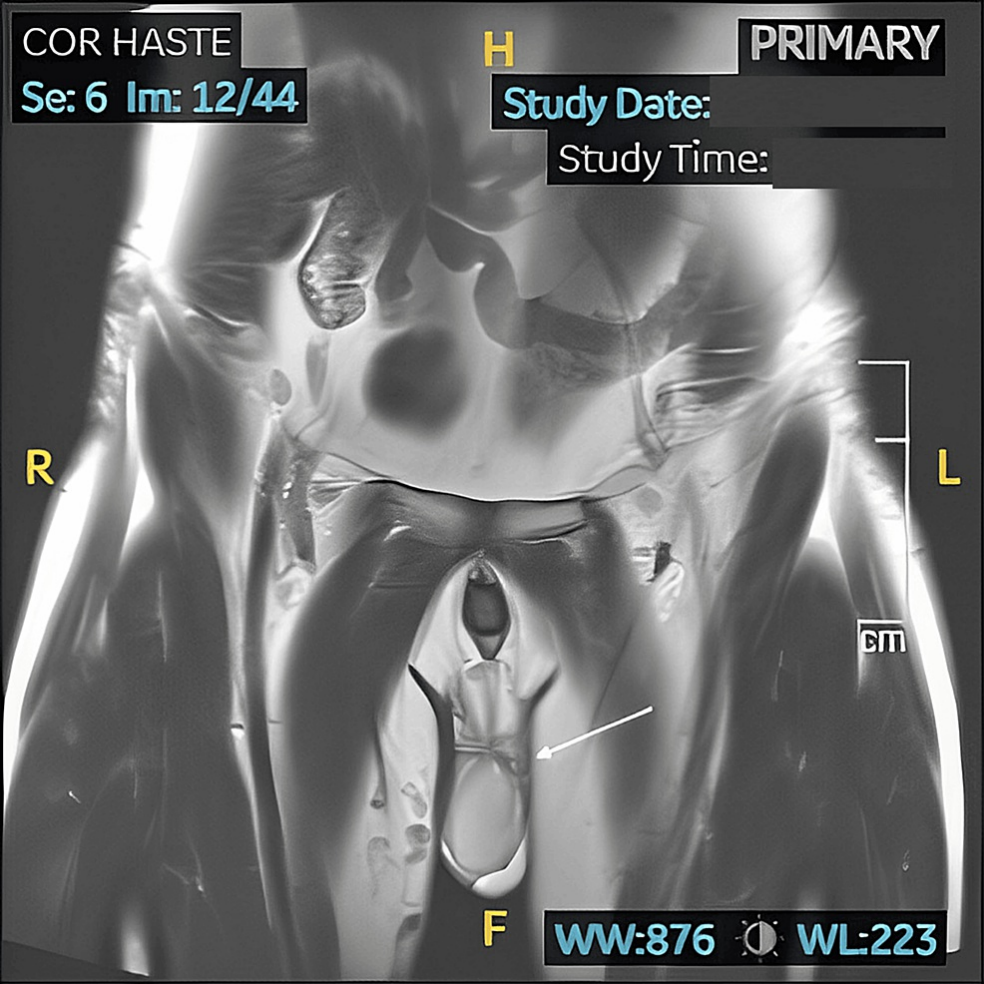
MRI of the pelvis in the coronal plane showing varicosities in the left hemiscrotum

**Figure 2:**
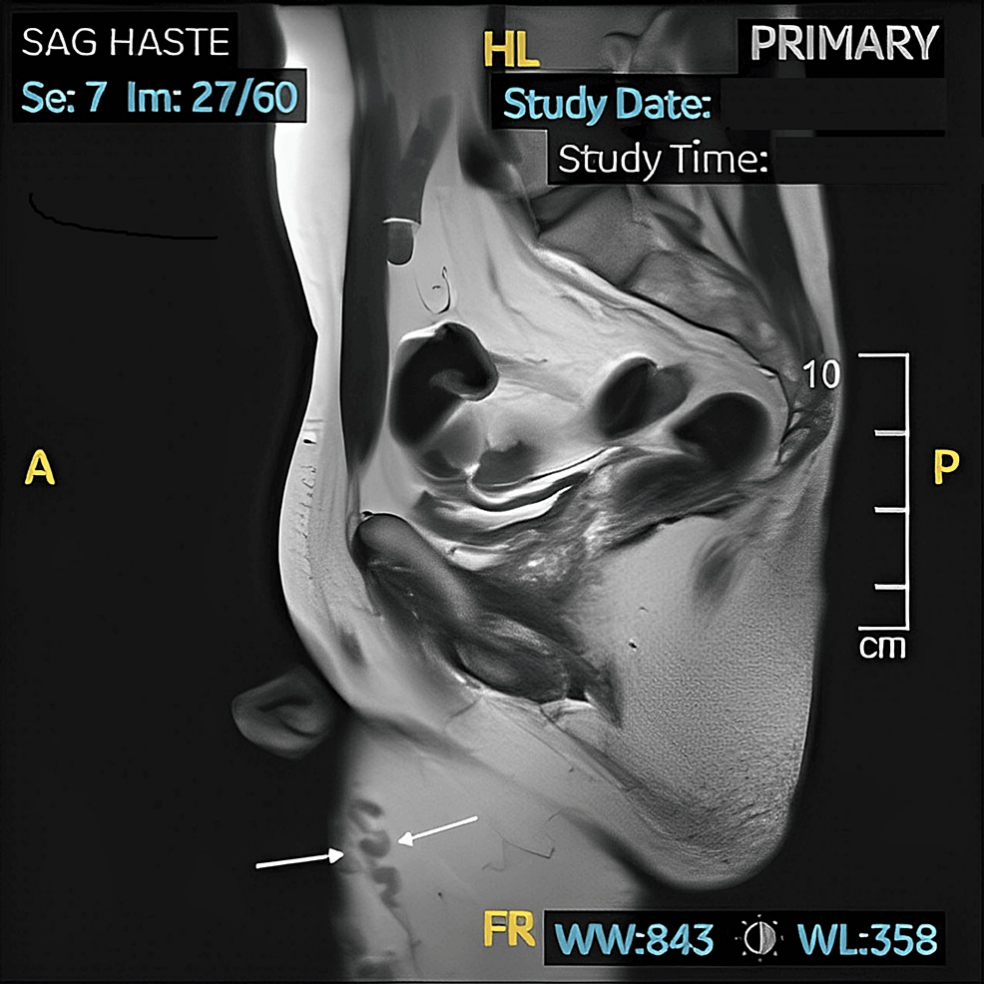
MRI of the pelvis showing venous varicosities seen in the anterior right thigh extending into the pelvis

Due to his persistent urinary frequency and urgency, he was diagnosed with overactive bladder (OAB). He was started on solifenacin and later switched to vibegron with mild symptom improvement. Due to symptom persistence, a cystoscopy was performed, revealing a mildly inflamed bladder neck, which was thought to be caused by bladder neck spasm post-ejaculation. A pelvic MRI was performed to assess for structural abnormalities, which showed varicosities extending from the right thigh into the pelvis (Figure 1) and left hemiscrotum suggestive of pelvic congestion (Figure 2). It was suspected that pelvic congestion may have contributed to the chronic pain, so interventional radiology was consulted to perform a pelvic venous embolization. After further discussion, the patient ultimately elected not to proceed with the embolization.

Neurology was subsequently consulted once urologic intervention failed to improve symptoms. Structural lesions, neuronal signaling deficits, and possible neuromuscular etiologies were assessed through MRI of the lumbar spine, somatosensory evoked potential test (SEP) of the pudendal nerves, and electromyography (EMG)/nerve conduction velocity (NCV) of the lower extremities. Pudendal SEP showed increased nerve latency suggestive of pudendal neuropathy. A pudendal nerve block relieved the pain for 48 hours, with repeat block emulating similar results. Unfortunately, the patient reported side effects of lower extremity numbness and falls. His gabapentin was titrated up to 600 mg three times a day (TID) with symptom improvement; however, the patient still experienced intermittent periods of debilitating pain with urinary frequency. With medical management failing to adequately control the symptoms of OAB and penile pain, surgical solutions were discussed. The patient consented to the surgical placement of an InterStim II device by Medtronic Inc. (Minneapolis, MN), which stimulates the sacral nerve. This procedure was conducted in two stages. In stage 1, the patient underwent a trial period during which he wore an external version of the device, enabling him to assess its impact on their symptoms. With the patient showing a positive response in stage 1, the urologist proceeded to stage 2, which involved the surgical implantation of the device.

At his one-month follow-up, the patient demonstrated the proper utilization of the device and reported significant improvements in both OAB and penile pain symptoms, rating his pain a 0/10. However, seven months after the device implantation, the patient unfortunately reported a return of his pain. He described this pain as occurring in his penis/urethra after ejaculation, and it was so severe that he even contemplated self-harm as a means to distract himself from it. The patient is currently maintaining regular follow-ups with his urologist and has been scheduled for a venogram to investigate potential underlying causes of the pain's recurrence. We are actively exploring additional medical and surgical treatment options that align with the patient's preferences.

## Discussion

Chronic penile pain is a rare condition, with an unknown incidence and prevalence [6]. Most men suffering from this condition undergo a lengthy evaluation from various specialists before finding a lasting treatment. Most patients who suffer from acute penile pain present with a clear etiology. However, in patients with chronic penile pain, the pathophysiology remains unknown. It is speculated that the dorsal nerve, a branch of the pudendal nerve (S2-S4) which is derived from the sacral roots, overlying the penis may be the primary culprit [6]. There have been few successful treatments noted for chronic penile pain, such as cryoablation and InterStim devices; however, the curative rate of both these treatments has not been well researched [6].

After two unsuccessful pudendal nerve blocks and failure of medical therapy (e.g., gabapentin) to provide adequate pain relief, the patient elected to have a sacral neuromodulation (SNM) procedure. SNM is a minimally invasive procedure, which is used to treat bowel and bladder dysfunctions [7]. The therapeutic technique uses electrical stimulation to sacral nerve roots S3 or S4. The therapeutic mechanism is not fully understood, but there are currently several working hypotheses, all of which are derived from the SNM possibly modulating the central and peripheral pathways of neural circuits that can override aberrant neural activity [7]. The mechanism of pain relief is speculated to be due to stimuli transmitted from SNM via Aβ‐fibers interfering with the c-fibers being stimulated by CPP [8].

Although SNM has not been FDA-approved for the treatment of CPP, both the European Association of Urology (EAU) and American Urological Association (AUA) guidelines suggest considering SNM for patients who have not responded well to medical interventions [8,9]. A recent systematic review and meta-analysis on the role of sacral neuromodulation in the management of CPP demonstrated that SNM could be an effective treatment option for reducing pain and improving patients' quality of life [8]. In the review, 26 out of the 1,026 identified articles were selected, evaluating 853 patients with CPP. Significant improvements in pain scores, ranging from 40% to 53%, were reported in 13 studies, and all studies reported an improvement in patients' quality of life, including a reduced need for medication use [8].

While our patient initially experienced pain relief after device implantation, it was reported during the seven-month follow-up that his pain returned with sexual activity, necessitating further evaluation. We are planning to consider future treatment options, both medical and surgical, that align with the patient's preferences. One significant limitation of this case report is that the management of the patient's penile pain is an ongoing process. Another limitation arises from the scarcity of data on the use of InterStim for chronic penile pain. Nevertheless, this case report emphasizes the pressing need for further research into management options for chronic penile pain and a deeper understanding of the physiological causes and etiology of these symptoms.

## Conclusions

There is insufficient data to support the best treatment approach for chronic penile pain; however, we have demonstrated that sacral neuromodulation systems, such as InterStim II, are potential treatment options for chronic penile pain refractory to medical therapy. Possible complications of this procedure include swelling, bruising, bleeding, pain at the implant site, new pain, infection, lead (thin wire) movement, technical or device problems, undesirable changes in urinary or bowel function, and uncomfortable stimulation. Our patient noted a significant decrease in penile pain following the InterStim II implant procedure without any reported complications. While the patient's pain returned seven months after the implantation, we are continuing to work with the patient to explore further treatment options. This case report highlights the complexity of this urogenital condition and the challenges that a primary care physician can encounter during the workup and management of patients with chronic penile pain. Due to the limited research on chronic penile pain, this case study contributes to the growing literature on this condition.
